# A turnaround strategy: improving equity in order to achieve quality of care and financial sustainability in Italy

**DOI:** 10.1186/s12939-018-0878-x

**Published:** 2018-11-20

**Authors:** Gianluca Cafagna, Chiara Seghieri, Milena Vainieri, Sabina Nuti

**Affiliations:** Health and Management Laboratory (MeS Lab), Institute of Management, Sant’Anna School of Advanced Studies, Piazza Martiri della Libertà, 24, Pisa, Italy

**Keywords:** Health equity, Socioeconomic status, Education, Financial sustainability, Quality of care, Heart failure, Italy, Performance management, Clinical pathway

## Abstract

**Background:**

Equity, financial sustainability, and quality in healthcare are key goals embraced by universal health systems. However, systematic performance management strategies for achieving equity are still weaker than those aimed at achieving financial sustainability and quality of care. Using a vertical equity perspective, the overarching aim of this paper is to examine how improving equity in quality of care impacts on financial sustainability. We applied a simulation to indicators of the heart failure clinical pathway in Tuscany (central Italy), in order to quantify the equity gaps and financial resources that could be reallocated in the absence of performance inequities.

**Methods:**

The analysis included all patients hospitalized for heart failure as a principal diagnosis in 2014. We selected five indicators: hospitalization rate, 30-day readmission, cardiology visits, and the utilization of beta-blockers, and ACE inhibitors and sartans. For each indicator, the simulation followed three steps: 1) stratification by socioeconomic status (SES), using education as a proxy for SES; 2) computation of the vertical equity indicator; and 3) assessment of the financial value of the equity gap.

**Results:**

All indicators showed performance gaps regarding inequities across SES-groups. For the hospitalization rate and 30-day readmission, resources could have been reallocated, if the performance of patients with a low SES had been equal to the performance of patients with a high SES, which amounted to €2,144,422 and €892,790 respectively. In contrast, limited additional resources would have been required for prescriptions and cardiology visits.

**Conclusions:**

Reducing equity gaps by improving the performance of low-SES patients may be a crucial strategy to achieving financial sustainability in universal coverage healthcare systems. Universal healthcare systems, which aim to pursue financial sustainability and quality of care, are thus urged to develop performance management actions to improve equity. This approach should not only include the measurement and public disclosure of equity indicators but be part of a comprehensive evidence-based strategy for the management of chronic conditions along the clinical pathway.

## Background

Equity, financial sustainability, and quality in healthcare are key goals embraced by universal health systems, and are recognized as overarching targets in several conceptual frameworks [1–3]. In order to measure the attainment of such goals, equity, financial sustainability, and quality indicators have been included in most performance evaluation systems (PESs). PESs evaluate performance units (e.g. nations, regions, local health authorities, hospitals, health districts) through benchmarking indicators in order to support stakeholders in defining objectives, targets, improvement strategies, and corrective actions [4, 5].

With regard to equity, Barsanti et al. [6] describe how horizontal and vertical equity [7] can be defined in performance management terms. In such terms, horizontal equity refers to geographical differences across performance units which cannot be explained by population needs. Improving horizontal equity therefore means reducing the unwarranted variation across performance units [8]. Possible horizontal equity targets for each performance unit are international, national or regional standards, or the average across performance units (if standards are unavailable). Vertical equity refers to socioeconomic (SES) inequities that cannot be explained by population needs, and improving vertical equity means reducing SES inequities in each local unit. A vertical equity target is the performance of the healthiest group, which is usually the one with the highest SES [6]. It is worth noting that the conceptualization of horizontal and vertical equity in performance management does not correspond to how they are traditionally defined within economics. In economics terms, vertical equity refers to the principle that groups and/or individuals with different care needs should be treated differently, whereas horizontal equity refers to the principle that groups and/or individuals with the same (or similar) care needs should be treated equally [9]. For the purposes of our discussion, we will refer to horizontal and vertical equity in performance management terms.

Post-industrialized countries, such as Great Britain and Italy, have increased efforts to promote horizontal equity [10–13]. They have implemented performance management actions aimed at reducing unwarranted geographical variations, such as systematic measuring and evaluation based on benchmarking data, public disclosure of these data, financial incentives, along with the publication of clinical guidelines. However, despite recent evidence from universal health systems showing that patients with a high SES still have better health outcomes and higher utilization of healthcare services than patients with a low SES [14, 15], systematic performance management strategies and governance systems to improve vertical equity are still weak. A recent study conducted in 10 European post-industrialized countries revealed that although equity has become a priority for most governments, limited performance management actions have been taken to reduce SES inequalities [16]. These actions are usually confined to specific projects, or limited to computing single indicators without adopting an integrated perspective. It is worth noting that the adoption of integrated care within a clinical pathway can contribute to equity improvements [17–19]. For example, a study carried out in Tuscany on the diabetic foot pathway concluded that a comprehensive strategy to improve equity should be carried out by favouring integration within the clinical pathway [19].

The aim of this paper is to examine the potential impact of improving equity in quality of care on financial sustainability. We applied a simulation to indicators of the heart failure (HF) clinical pathway in Tuscany, in order to quantify the equity gaps and financial resources that could be reallocated to services with a higher value for patients, in the absence of performance inequities.

Previous literature on the relationship between equity and health systems goals related to financial sustainability, such as efficiency, remains inconclusive. A number of researchers from the economics literature emphasized the possibility of equity-efficiency trade-offs in health and health care [20–23]. Given these conflicts between equity and efficiency, scholars stressed the importance of assigning explicit weights to each goal in priority setting [24]. More recent evidence questions the traditional claim on the equity-efficiency trade-off [25–27]. For example, Culyer claimed that efficiency and equity are not inherently in conflict and that an *“inefficient allocation can become more efficient without increasing inequity”* [26]. With regards to performance measurement, evidence on the equity-efficiency trade-off is limited and inconclusive. For example, Davis el al. found scarce congruity in the rankings of hospitals across the equity, efficiency and effectiveness dimensions [28], whereas the Commonwealth Fund showed a consistent relationship between how countries perform in terms of equity and efficiency (the higher the equity performance, the higher the efficiency performance) [29]. It is worth noting these results are influenced by which measures of equity/efficiency were selected and how the rankings were calculated.

Our study contributes more directly to the literature assessing the impact of reducing SES inequalities on financial sustainability. Previous evidence calculated the economic burden of SES inequalities in health and health care, suggesting that reducing SES inequalities can favour financial sustainability [30–33]. In order to calculate the economic burden of SES inequalities, these studies assumed that lower SES groups have the same average costs [30, 31] or the same health attainment [32, 33] as the highest SES group.

The main element of novelty introduced by this study is its performance management perspective. Our simulation assumed that the lowest SES group had the same performance as the highest SES group, in order to determine how much resources could be potentially reallocated. Note that the performance of the group with the highest SES can be considered as a vertical equity target in performance management terms.

We selected Tuscany as a setting for carrying out this study because it is region of a country with a universal healthcare system (i.e. Italy) and has considerable experience in performance management. The Italian National Health System (NHS) provides universal healthcare coverage to all individual residents and should ensure equity by mandate. It is financed mainly through general taxation, supplemented by co-payment for outpatient care and medicines. Public sources account for 78.2% of total health expenditure and out-of-pockets payment for 17.8% [34]. Private health insurance plays a supplementary role, as it reaches only 1% of the Italian total health expenditure [35].

In 2005, the Management and Health Laboratory of the Sant’Anna School of Advanced Studies developed a multidimensional PES to monitor and assess local health authorities (LHAs) in Tuscany [36, 37]. LHAs are geographically and population-defined health institutions, which are financed per capita by Tuscany [34]. LHAs are subject to performance assessment, as they are directly responsible for the organization and delivery of healthcare services to their corresponding population, including at the clinical pathway level. The Tuscan PES benchmarks LHAs through indicators organized into six performance dimensions (i.e. health outcome, ability to pursue regional strategies, quality of health and social care, staff satisfaction, patient experience, efficiency and financial sustainability), using data from administrative sources and surveys of patients and healthcare employees. Over the last 10 years, the Tuscan PES has been adopted by another 12 regions in Italy [38]. Vertical equity indicators were included in the Tuscan PES in 2010.

This study focuses on the HF clinical pathway for two main reasons. First, HF is an ambulatory care-sensitive condition (ACSC). Hospitalizations for ACSCs are considered preventable through the quality of care achieved by early diagnosis and treatment at the primary care level and adequate disease management, and have been shown to be related to patients’ SES [39–41]. Second, the HF clinical pathway is relevant in terms of financial sustainability. HF is a chronic condition with the highest hospital discharge diagnosis and has a higher than 50% expected one-year readmission [42, 43].

## Method

### Data

We conducted an explanatory simulation using administrative data. The analysis included all patients hospitalized in Tuscany in the 2014 year for HF as the principal diagnosis, as identified by the Italian National Outcome Program [44]. We included all patients hospitalized with the International Classification of Diseases, 9th revision, Clinical Modification (ICD-9-CM) codes 398.91, 402.01, 402.11, 402.91, 404.01, 404.03, 404.11, 404.13, 404.91, 404.93, 428.0, 428.1, 428.2, 428.3, 428.4, 428.9. For each patient, administrative databases in Tuscany enabled us to use socio-demographic variables (age, sex and SES), clinical variables (diagnosis and comorbidities) and cost-related variables (diagnosis-related group (DRG) tariffs, medical visits tariffs, and prescription medication prices). We also used the last available population data from the 2011 Italian Population and Housing Census, in order to compute the hospitalization rate. A unique identifier was assigned to each patient by the office of the Regional Health Information System and enabled record linkage of administrative databases in Tuscany. The identity of patients or other sensitive data are not disclosed by the identifier, thus ensuring compliance with Italian privacy laws. Given that we used administrative data, an ethics committee approval was unnecessary.

### Indicators included in the simulation

We included indicators based on the Tuscan PES that are relevant for equity analyses [15, 45–48]. These indicators reflect the quality of care provided by LHAs in a clinical pathway perspective. An appropriate clinical pathway strategy to manage chronic conditions, such as HF, should be based on a timely and effective diagnosis and treatment in the primary care setting and appropriate disease management (e.g. compliance with pharmacological treatments and routine cardiology visits for HF). Hospitalizations however, should be avoided when possible, because they are both costly for health systems and risky for patients’ health. Evidence suggests that hospitalization for ACSC can be avoided through the adoption of an appropriate and coordinated clinical pathway strategy [49].

The indicators selected were: hospitalization rate, 30-day readmission, utilization of beta-blockers, utilization of angiotensin-converting enzyme (ACE) inhibitors and sartans, and cardiology visits. For beta-blockers, ACE inhibitors and sartans, and cardiology visits, we identified the same cohort of patients and followed them for one year after their discharge. The cohort was identified by excluding patients who did not reside in Tuscany, patients who had died during hospitalization, or who had been discharged by non-accredited private hospitals. For the hospitalization rate and 30-day readmission, we excluded patients based on the same criteria used in the Tuscan PES. Note that excluding private hospitals does not affect our results, given that only 0.14% of hospitalizations for HF were in private hospitals. The HF hospitalizations correspond to 1.33% of all hospitalizations in Tuscany with the same selection criteria.

The aim of this study was not to comprehensively assess the potential opportunities for resource reallocation in the HF clinical pathway through these indicators. The selected indicators were simply used as examples to identify areas where a reduction in inequities could generate opportunities for resource reallocation. Table 1 shows the definition, design, and method used to assess the financial value of each indicator selected.

**Table 1:** Definition, design, and variables used to assess financial value of each selected indicator

Indicator	Definition	Design	Method used to assess financial value
Hospitalization rate	Number of hospital admissions for HF/Number of residents * 100,000	Cross-section	DRG tariffs
30-day readmission	Unplanned rehospitalisation occurring for any cause within 30 days of discharge for HF	Cohort	DRG tariffs
Utilization of Beta-blockers	Average consumption of beta blockers within one year after discharge for HF	Cohort	Price of beta-blockers
Utilization of ACE inhibitors and sartans	Average consumption of ACE inhibitors and sartans within one year after discharge for HF	Cohort	Price of ACE inhibitors and sartans
Cardiology visits	Average number of cardiology visits within one year after discharge for HF	Cohort	Cardiology visit tariffs

### The simulation

For each selected indicator, the simulation followed three steps: 1) stratification by SES, 2) computation of the vertical equity indicator, and 3) assessment of the financial value of the equity gap.

The first step was to stratify each indicator by SES for each LHA. We used the individual educational level as a proxy for SES [50], and categorized patients in two groups: patients with low education (middle school diploma or less) and patients with high education (high school diploma or more). This categorization is consistent with previously published studies [6] and replicates the Tuscan PES categorization for equity indicators [51]. In line with other evidence, patients whose level of education had not been recorded (13.56%) were excluded from the analysis [14, 52]. Performance indicators were computed for both patients with a low and high education.

In line with related evidence [53], we standardized performance indicators using the indirect method [54], in order to control for differences in age, sex and comorbidities at the LHA level. We thus preferred to use the term “equity” and not “equality” when referring to this study. Note that inequalities refer to differences, whereas inequities refer to inequalities (i.e. differences) that can be considered as “avoidable” or “unjust”. The main difference between inequities and inequalities is the involvement of a moral judgement [9].

With regard to comorbidities, we computed the Charlson index (CI) based on three years prior to hospitalization for HF. The CI is considered a reliable method to assess comorbidities and has been validated for both hospitals and primary care settings [42, 55]. The second step was to compute the vertical equity indicator corresponding to each selected indicator for each LHA. A vertical equity indicator is equal to the ratio between the performance indicator for patients with low SES and the performance indicator for patients with high SES. If the ratio is equal to 1, there are no SES inequities; the further the ratio is from 1, the higher the SES inequities.

The third step was to assess the financial value of the equity gap for each indicator for each LHA. In other words, we computed the financial resources that could be reallocated if zero SES inequities in quality and outcome performance indicators were achieved. The potential resources to be reallocated in absolute terms refer to how many resources (€) each LHA could have reallocated if the performance of patients with a low SES had been equal to the performance of patients with a high SES. The potential resources to be reallocated in relative terms refer to the total percentage of resources used for patients with a low SES that each LHA could been have reallocated, if the performance of patients with low a SES had been equal to the performance of patients with a high SES. For example, in order to express the financial value of the equity gap for cardiology visits, we computed for each LHA: a) Number of cardiology visits within one year after discharge for patients with low education if they had performed in the same way as patients with high education (number of low-educated patients * average number of cardiology visits within one year after discharge for high-educated patients); b) Surplus/deficit of cardiology visits for low-educated patients (number of cardiology visits one year after discharge for low-educated patients - (a)); c) Financial value of the equity gap ((b) * average cost of a cardiology examination for low-educated patients).

The assessment of financial values was based on a previous work conducted by Nuti el al., which quantified how much budget could have been reallocated in Tuscany if LHAs had achieved the regional performance average or the best performance [56]. Table 2 provides details on sample size and patients’ characteristics (i.e. age, sex, CI, and level of education) for cardiology visits as an example. We carried out all analyses using SAS for Windows, version 9.3 (SAS Institute, Cary, NC) and STATA, version 13 (StataCorp LP, College Station, TX).

**Table 2:** Cardiology visits: baseline characteristics of study population, stratified by LHAs

LHA*	Number of observation	Age (years)	Male (%)	CI > 0 (%)	High education (%)
1	393	80.33	49.62%	37.66%	8.14%
2	196	78.29	52.55%	52.55%	6.63%
3	787	81.12	45.49%	42.82%	6.73%
4	472	81.47	49.36%	43.64%	5.08%
5	543	79.90	46.96%	35.91%	12.52%
6	640	79.87	51.41%	39.53%	12.81%
7	606	80.02	57.59%	38.12%	12.38%
8	626	80.91	49.36%	45.05%	7.03%
9	210	78.82	49.05%	38.57%	15.71%
10	1209	80.35	49.55%	44.50%	7.78%
11	539	80.25	45.45%	38.96%	7.79%
12	236	76.19	58.05%	44.92%	8.90%
Tuscany	6457	80.19	49.79%	41.66%	9.00%

Notes: * Age (years), male (%), CI > 0 (%), and high education (%) are significantly different among LHAs (*p* < 0.001)

## Results

### Hospitalization rate

For all LHAs, the hospitalization rate for patients with a low SES was higher than the hospitalization rate for patients with a high SES. The vertical equity indicator for Tuscany was 2.86. These inequities were reflected in the potential resources to be reallocated for all LHAs. If the hospitalization rate for patients with a low SES had been equal to the hospitalization rate for patients with a high SES, there would have been an additional €2,144,422 to be reallocated in Tuscany, which corresponds to 65.22% of the resources used for the hospitalization of low-SES patients. The percentage of resources to be reallocated ranged from 30.40 to 78.99%. Table 3 shows the results for hospitalization rate.

**Table 3:** Hospitalization rate

LHA	Vertical equity indicator	Potential resources to be reallocated	Potential resources to be reallocated
		Absolute terms (€)	Relative terms (%)
1	2.83	71,062.18	55.29
2	3.01	29,768.99	30.40
3	2.98	373,285.28	78.99
4	2.79	144,914.27	63.25
5	2.78	151,681.74	60.55
6	3.19	241,768.22	70.77
7	2.66	216,712.20	68.90
8	3.07	219,415.03	69.68
9	2.69	27,419.79	28.99
10	2.76	347,118.70	61.19
11	2.48	199,255.91	67.25
12	3.37	133,388.87	74.02
Tuscany	2.86	2,144,422.22	65.22

### 30-day readmission

Patients with a low SES had a higher 30-day readmission than patients with a high SES, for all LHAs except for two. For both exceptions, the vertical equity indicator was relatively close to one, meaning that the 30-day readmission was similar for both SES groups. The potential recourses to be reallocated in the absence of performance inequality for Tuscany would be €892,799, which corresponds to 26.82% of the resources used for the 30-day readmission for patients with a low SES. The percentage of resources to be reallocated ranged from 14.17 to 71.17%, considering LHAs with a vertical equity indicator higher than one. Table 4 shows the results for 30-day readmission.

**Table 4:** 30-day readmission

LHA	Vertical equity indicator	Potential resource to be reallocated	Potential resource to be reallocated
		Absolute terms (€)	Relative terms (%)
1	0.92	-20,205.00	−9.46
2	1.62	69,849.07	42.84
3	1.33	104,712.90	25.23
4	3.48	177,871.50	71.17
5	0.88	−38,108.05	−16.57
6	1.31	71,643.42	22.53
7	1.43	81,655.66	29.64
8	1.20	57,404.49	19.31
9	1.17	10,476.55	14.17
10	1.77	283,720.66	44.14
11	1.26	42,974.97	16.84
12	3.49	138,118.22	70.97
Tuscany	1.37	892,799.73	26.82

Notes: The minus sign indicates additional resources for spending

### Utilization of beta-blockers

Low-SES patients consumed fewer beta-blockers than high-SES patients in all LHAs except two. The vertical equity indicators for beta-blockers were closer to 1 (0.92 for Tuscany), suggesting that performance inequities across SES-groups in terms of beta-blocker utilization are relatively weak. Tuscany would have required an extra €15,810 in the absence of performance inequities across SES-groups, which corresponds to 8.38% of the resources for beta-blockers of low-educated patients. The percentage of potential resources to be reallocated ranged from 0.52 to 28.77%, considering LHAs with a vertical equity indicator lower than 1. Table 5 shows the results for utilization of beta-blockers.

**Table 5:** Utilization of beta-blockers

LHA	Vertical equity indicator	Potential resources to be reallocated	Potential resources to be reallocated
		Absolute terms (€)	Relative terms (%)
1	0.80	− 3018.83	−25.64
2	0.98	−231.21	−3.05
3	1.03	561.07	2.32
4	1.13	1131.81	8.78
5	0.98	−79.53	−0.52
6	0.92	− 1271.62	−7.39
7	0.82	− 2297.62	−24.75
8	1.00	− 383.58	−2.08
9	0.85	− 883.72	−16.66
10	0.87	− 6581.68	−15.45
11	0.77	− 4666.00	−28.77
12	0.84	− 563.88	−7.16
Tuscany	0.92	−15,810.72	−8.38

Notes: The minus sign indicates additional resources for spending

### Utilization of ACE inhibitors and sartans

The average consumption of ACE inhibitors and sartans within one year after discharge for HF was lower for patients with a low SES, for all LHAs except three. The vertical equity indicator for Tuscany was 0.84. If patients with a low SES had reached the average consumption of ACE inhibitors and sartans of patients with a high SES, Tuscany would have required €46,706 extra, which corresponds to 19.04% of the resources spent for low-educated patients. When focusing on LHAs with a vertical equity indicator lower than 1, the percentage of resources to be reallocated varied from 12.01 to 53.59%. Table 6 shows the results for utilization of ACE inhibitors and sartans.

**Table 6:** Utilization of ACE inhibitors and sartans

LHA	Vertical equity indicator	Potential resources to be reallocated	Potential resources to be reallocated
		Absolute terms (€)	Relative terms (%)
1	0.66	− 7254.33	−53.59
2	0.79	− 1769.29	−23.49
3	0.78	− 9940.97	−30.22
4	1.52	4913.36	31.66
5	1.16	3747.67	16.58
6	0.87	− 2850.44	−12.01
7	0.73	− 8022.62	−40.07
8	0.75	− 8555.76	−37.99
9	0.80	− 2023.87	−23.44
10	0.84	− 9007.03	−19.57
11	0.75	− 6557.97	− 28.92
12	1.13	2014.55	20.83
Tuscany	0.84	−46,706.89	−19.04

Notes: The minus sign indicates additional resources for spending

### Cardiology visits

The average number of cardiology visits within one year after discharge for HF was higher for patients with a high SES, for all LHAs except four. This implies that 8 out of 12 LHAs would have spent more resources if low-SES patients had reached the average number of cardiology visits as high-SES patients. Tuscany would have required €24,431 extra in the absence of performance inequities, which corresponds to 25.68% of the resources used for cardiology visits of low-educated patients. The variation in the potential additional resources for spending was high, ranging from 15.99 to 80.11%, when focusing on LHAs with a lower vertical equity indicator than 1. Table 7 shows the results for cardiology visits.

**Table 7:** Cardiology visits

LHA	Vertical equity indicator	Potential resources to be reallocated	Potential resources to be reallocated
		Absolute terms (€)	Relative terms (%)
1	1.69	1299.78	37.10
2	1.02	96.72	2.06
3	0.72	− 5237.47	−48.91
4	0.89	− 1208.81	−24.20
5	0.73	− 3606.33	−41.61
6	0.90	− 1569.89	−15.99
7	0.82	− 2018.71	−31.59
8	0.67	− 4741.03	−61.95
9	1.17	292.73	9.51
10	1.07	166.43	0.78
11	0.68	− 5314.66	−51.74
12	0.50	− 3224.43	−80.11
Tuscany	0.84	−24,431.08	−25.68

Notes: The minus sign indicates additional resources for spending

To illustrate the methodology behind our calculations, we show how we assessed the financial value of the equity gap by using cardiology visits as an example. As stated in the method section above, we computed: a) Number of cardiology visits within one year after discharge for patients with low education if they had performed in the same way as patients with high education (n ≈ 3120.62), which is equal to the number of low-educated patients (*n* = 5870) * average number of cardiology visits within one year after discharge for high-educated patients (n ≈ 0.53); b) Deficit of cardiology visits for low-educated patients (n ≈ − 637.62), which is equal to number of cardiology visits one year after discharge for low-educated patients (*n* = 2483) - (a: n ≈ 3120.62); c) Financial value of the equity gap (≈ − €24,431.08), which is equal to (b: ≈ − 637.62) * average cost of a cardiology examination for low-educated patients (≈ €38.32).

## Discussion

This simulation highlighted areas of the HF clinical pathway in which a reduction in performance inequities could generate opportunities for resource reallocation. The hospitalization rate and 30-day readmission were higher for patients with a low SES relative to patients with a high SES (the vertical equity indicator was higher than 1). This is in line with previous evidence suggesting that hospitalizations for ACSC are strongly influenced by SES [39–41]. The reasons behind these SES inequities include lower self-management capacity, understanding of physicians’ recommendations, and the compliance with therapeutic measures of low-SES patients compared to high-SES patients [57–59]. Given that the SES inequities for hospitalization rate and 30-day readmissions were reflected in the potential resources to be reallocated, there is a financial case for tackling these inequities.

Cardiology visits, beta-blockers, and ACE inhibitors and sartans indicators also presented SES inequities. Their patterns of use were lower for low-SES patients than high-SES patients. These patterns cannot be directly explained by co-payments in Italy because HF patients are exempt of co-payments for healthcare services and medicines related to HF (Ministerial Decree no. 329 of 1999 and no. 296 of 2001). Cardiology visits are usually scheduled after discharge and managed by a general practitioner (GP), which act as a gate-keeper in the Italian healthcare system [34]. Previous evidence has shown that the low-SES patients visit their GP more frequently than high-SES patients [15, 60]. However, they are less likely to be visited by a specialist than high-SES patients; therefore, it can be reasonable that our results are related to a lower self-management capacity and/or limited awareness of co-payment exemptions of low SES patients with HF.

On a related note, the recommended medications to reduce the mortality risk of patients with heart failure, such as beta-blockers and statins, have been shown to be prescribed less often to patients with a low SES [46, 61]. In contrast to our results, a recent systematic review found higher prescriptions of ACE inhibitors to the lowest SES groups. However, the same study highlighted that the reasons behind the variation in prescriptions are still unclear [46]. It is worth noting that beta-blockers and ACE inhibitors are complementary, which might justify some differences in their pattern of use [62].

Unlike the hospitalization rate and 30-day readmission, a reduction in SES inequities would result in additional spending resources for cardiology visits, beta-blockers and ACE inhibitors and sartans. In fact, low-SES patients should increase their utilization of medications and visits, in order to reduce performance inequities. For cardiology visits, beta-blockers and ACE inhibitors and sartans, although there is room for improvement in terms of SES inequities, there is no financial case for tackling them.

Considering all the selected indicators from a clinical pathway perspective, the potential additional resources for spending on cardiology visits, beta-blockers, and ACE inhibitors and sartans are limited in relation to the potential resources that could be reallocated for the hospitalization rate and 30-day readmissions (Table 8). The highest opportunity for resources reallocation both in absolute and relative terms concerned the hospitalization rate. There would be additional €2,057,475 that could be reallocated in Tuscany, if the performance of low-SES patients reached the same performance as high-SES patients in all our indicators expect 30-day readmission. We excluded the potential recourses that could be reallocated for 30-day readmission, as they are already captured by the hospitalization rate. We estimated that €2,057,475 corresponds to the 20.38% of all the resources spent for the HF patients included in our study (€10,091,355). This last rough figure is derived by multiplying a unitary annual cost per HF patient (€870.98), which includes all outpatient visits, medicines, and Chronic Care Model (CCM) services related to the HF condition (for details, see Nuti and Vainieri, 2013 [63]) by the number of HF patients in our sample, and then adding the total cost of hospitalizations for HF patients in our sample.

**Table 8:** Vertical equity indicators and potential resources to be reallocated in Tuscany

LHA	Vertical equity indicator	Potential resources to be reallocated	Potential resources to be reallocated
		Absolute terms (€)	Relative terms (%)
Hospitalization rate	2.86	2,144,422.22	65.22
30-day readmission	1.37	892,799.73	26.82
Cardiology visits	0.84	−24,431.08	− 25.68
Utilization of Beta-blocker	0.92	−15,810.72	−8.38
Utilization of ACE inhibitors and sartans	0.84	−46,706.89	−19.04

Notes: The minus sign indicates additional resources for spending

### Policy and performance management implications

Improving the performance of patients with a low education can contribute to three key goals of universal health systems: 1) equity, by reducing the gap between patients with low and high education; 2) quality of healthcare, by following clinical protocols and ensuring the delivery of timely and appropriate care to patients with low education; and 3) financial sustainability, by generating opportunities for resource reallocation within a clinical pathway, as suggested by our results.

Therefore, the main implication of our study is that policy makers and LHAs managers should stop considering the performance of low-SES groups as a minor and separate concern, as efforts to improve equity do more than benefit equity alone. In fact, improving the performance of low-SES groups provides a valuable opportunity to improve financial sustainability by generating additional resources within the clinical pathway.

From a policy perspective, the results of this study suggest that efforts should be directed towards improving self-management capacity, understanding of doctor recommendations, and compliance with therapies of low-SES patients. These efforts should favour a more adequate use of cardiology visits, beta-blockers and ACE inhibitors and sartans by low-SES patients, which result in a reduction of their avoidable hospitalizations. Examples of recommended evidence-based interventions for the management of chronic conditions include post-hospitalization support for low-SES patients [64], patient-centred adherence interventions [65] and the CCM. In 2010, Tuscany implemented a CCM for patients with chronic diseases (e.g. HF, stroke and chronic obstructive pulmonary disease, type 2 diabetes) in order to favour a shift from an acute, episodic, and reactive care, to a preventive, integrated, and proactive one [66]. Evidence suggest that the CCM contribute to better patient outcomes, quality of care, and financial sustainability [18, 66, 67].

From a performance management perspective, our results suggest to increase efforts towards developing an effective strategy for vertical equity. The first step of such a strategy is the systematic measurement and public disclosure of vertical equity indicators, as they contribute to raising awareness about equity gaps and motivating policy actions [6, 19, 68, 69]. In Tuscany, the adoption of a PES based on benchmarking, a performance visualization tool (i.e. dashboard) and the public disclosure of information led to performance improvements in most indicators, and a reduction in the unwarranted geographical variations over time [37].

In addition, the measurement and public disclosure of vertical equity should not be limited to specific indicators or projects but a clinical-pathway perspective should be adopted instead. This means that SES inequities should be assessed from the earliest stages and across different areas in pathways. A good example is the monitoring framework of the British NHS, which includes *“monitoring equity at all main stages of the patient pathway”* among its design objectives [70]. The adoption of a clinical-pathway perspective provides a way to better define proactive policies as close as possible to patients’ SES [42].

Finally, equity performance measurement can be combined with evidence-based interventions for the management of chronic conditions, such as the CCM [42]. Equity performance measurement should thus be considered as part of a comprehensive evidence-based strategy for the management of chronic conditions along the clinical pathway. Buja et al. recently called for creative solutions to address the burden of chronic conditions and supported a proactive approach to chronic care [42]. The systematic measurement of vertical equity at all the principal stages of the clinical pathway can contribute to a more accurate identification of patients who are most in need [70] and, thus, to the proactive management of chronic conditions. On the basis of our results, it seems reasonable to argue that healthcare organizations applying CCM may achieve even better results, if they start by measuring vertical equity and promoting a proactive management of the most socio-economically disadvantaged patients.

### Comparisons with other studies

Previous studied related to the impact of reducing SES inequalities on financial sustainability have attempted to measure the costs of both healthcare and heath inequalities. Overall, existing evidence is aligned with our study, as it confirms that there is a financial case for tackling SES inequalities [30].

Regarding the cost of healthcare inequalities, Asaria et al. [30] quantified the hospital care costs of SES inequalities in the British NHS, using small-area-level deprivation as a SES variable. The cost of SES inequalities associated with hospital admissions was £4.8 billion in 2011/2012 [30]. Interestingly, the study used survival models to estimate cumulative lifetime costs, concluding that the higher healthcare costs of low-SES patients outweigh the reduction in healthcare costs due to their lower life expectancy [30].

Similar to our study, Dimitrovová et al. [39] estimated that the annual cost of inequalities in hospitalization rates for ACSC was more than €15 million in the Portugal. These authors used area-based illiteracy and purchasing power as SES indicators.

The Public Health Agency of Canada (PHAC) computed the burden of SES inequalities for acute-care hospitalizations, prescription medications and medical consultations, using income as a proxy for SES [31]. The study concluded that SES inequalities cost Canada $6.2 billion annually. In contrast to our results, the PHAC found opportunities for resource reallocation in prescription medications and medical consultations, not only in hospitalizations. This difference might be due to our focus on the HF clinical pathway. For example, with respect to medical consultations, our study focused only on cardiology visits. Previous evidence has shown that patients with a low SES are more likely to be seen by their GP, but less likely to have specialist visits than those with a high SES, thus possibly explaining this difference in results [15, 60]. On a similar note, we focused only on beta-blockers and ACE inhibitors/sartans, whereas the PHAC focused on all prescription medications.

All the previously mentioned studies on the cost of healthcare inequalities were conducted in countries that have universal healthcare coverage like Italy. However, unlike our study, they all used area-based SES measures, which might have increased the risk of ecological fallacy.

As to the cost of health inequalities, a European Commission study estimated that if all the population had the same health status as those with a higher education, this would decrease hospitalizations and GP visits, resulting in a cost reduction of approximately €85 billion per year [32]. Similarly, a United States study concluded that eliminating health disparities between the white population and minorities (African Americans, Asians and Hispanics) would result in a decrease of about $230 billion over 2003–2006 [33].

None of the studies mentioned adopted a performance management perspective. Our simulation differs from the other studies because it used a vertical equity performance target (i.e. the performance of the group with the highest SES) as a counterfactual, providing evidence of the potential financial gains if all LHAs achieve the target. In other words, to compute the potential resources to be reallocated, we assumed that the lowest SES group had the same performance as the highest SES group for each LHA.

In contrast, studies on the cost of healthcare inequalities usually assume that lower SES groups have the same average costs as the highest SES groups, while studies on the costs of health inequalities assume that lower SES groups have the same health attainment as the highest SES groups [31]. Note that all these studies use the highest SES group as a counterfactual. It is important to specify that the performance of the highest SES group can be considered as a vertical equity target [6], and then used as a counterfactual, because equity analyses both in health and health care usually show that high-SES patients experience better health outcomes and receive more appropriate healthcare services than low-SES patients [40–42, 61]. This consistent trend in the literature is behind an important assumption of our simulation: the use of healthcare services by high-SES patients is more appropriate than the use by low-SES patients. For example, our simulation implicitly considers more likely that HF patients with a low SES under-consume beta-blockers than HF patients with a high SES over-consume them.

### Strengths and limitations

We believe that this is the first study on the cost of healthcare inequities that has adopted a performance management perspective within a clinical pathway. This enabled us to provide innovative performance management implications, which go beyond just computing the financial burden of healthcare inequities. Secondly, unlike recent studies on the cost of healthcare inequalities [30, 31], we were able to control for differences in age, sex, and comorbidities at the LHA level using indirect standardization. This standardization allowed us be consistent with the indicators of the Tuscan PES and provide data- and context-based performance management implications. It is worth noting that our results and key messages hold regardless of the presence or absence of significant differences in the vertical equity indicators across LHAs. In either case, eliminating performance inequities would free up resources in Tuscany. In contrast, providing a vertical equity ranking of LHAs is out of the scope of this study. Thirdly, we avoided the risk of ecological fallacy by using individual-level education data; whereas several studies have used area-based SES variables due to the lack of data at the individual level [30, 31, 39]. Note that a previous study conducted in Italy considered education data in hospital discharge records as fairly reliable and valid [71].

One limitation of our study is that because of the administrative nature of our data, we were unable to detect all patients with HF in Tuscany, thus, our analysis focused on all patients hospitalized for HF as the principal diagnosis in 2014. As a consequence, it is likely that the potential resources to be reallocated are an underestimation. However, our selection is commonly used for PES indicators and is aligned with the performance management perspective of this study.

Another limitation is that we used DRG tariffs as a method to assess the financial value of the hospitalization rate and 30-day readmission. A DRG tariff is not a direct measure of costs related to hospitalization, as it is an all-inclusive remuneration fee associated with the average treatment of a hospitalization category. This might have introduced biases; however, the DRG tariff was our best available proxy to measure the financial value of the hospitalization rate and has been used in similar studies [39, 56]. Other financial assessment methods can be found in Nuti et al. (2010) [56].

Although there was some overlap in the potential resources to be reallocated across the 30-day readmission and the hospitalization rate, differentiating between these two indicators have enabled us to better identify priorities within the HF clinical pathway [56]. For example, if SES inequities and related resources to be reallocated had mainly concerned the 30-day readmission, policy makers should prioritize post-hospitalization assistance to patients with a low SES [14].

While we used only individual-level education as a proxy for SES due to the lack of other reliable and valid SES variables, education is widely recognized as a good proxy for SES [50]. However, including SES variables other than education would have provided a broader picture of SES.

## Conclusions

This simulation provided evidence of the impact of improving vertical equity on financial sustainability in the HF clinical pathway in Tuscany. Our results showed that if the performance of patients with a low-SES were equal to the performance of patients with a high-SES, there would be resources that could be freed up for hospitalization rate and 30-day readmission, whereas limited additional resources would be required for prescriptions and cardiology visits.

Universal health systems, which aim to pursue equity, quality of health care, and financial sustainability are thus urged to develop performance management actions to improve vertical equity. These actions should move beyond the measurement and public disclosure of vertical equity indicators and be part of a comprehensive evidence-based strategy for the management of chronic conditions along the clinical pathway and for the commitment of healthcare professionals to the improvement process.

We recommend that further research should use whole-population data to comprehensively assess the impact of reducing SES inequities on financial sustainability along clinical pathways. Research is also needed to develop a comprehensive strategy for the management of chronic conditions, which include performance management actions for equity.

## Abbreviations

ACE: Angiotensin-converting enzyme
ACSC: Ambulatory care-sensitive condition
CCM: Chronic Care Model
CI: Charlson index
DRG: Diagnosis-related group
GP: General practitioner
HF: Heart failure
ICD-9-CM: International classification of diseases, 9th revision, clinical modification
LHA: Local health authority
NHS: National Health Service
PES: Performance evaluation system
PHAC: Public Health Agency of Canada
SES: Socioeconomic status
